# Changes in corticospinal drive to spinal motoneurones following tablet‐based practice of manual dexterity

**DOI:** 10.14814/phy2.12684

**Published:** 2016-01-26

**Authors:** Lisbeth H. Larsen, Thor Jensen, Mark S. Christensen, Jesper Lundbye‐Jensen, Henning Langberg, Jens B. Nielsen

**Affiliations:** ^1^ CopenRehabDepartment of Public HealthUniversity of CopenhagenCopenhagen KDenmark; ^2^ Department of Nutrition, Exercise and SportsUniversity of CopenhagenCopenhagen NDenmark; ^3^ Department of Neuroscience and PharmacologyUniversity of CopenhagenCopenhagen NDenmark

**Keywords:** Tablet‐based practice, corticomuscular, coherence, plasticity, manual dexterity

## Abstract

The use of touch screens, which require a high level of manual dexterity, has exploded since the development of smartphone and tablet technology. Manual dexterity relies on effective corticospinal control of finger muscles, and we therefore hypothesized that corticospinal drive to finger muscles can be optimized by tablet‐based motor practice. To investigate this, sixteen able‐bodied females practiced a tablet‐based game (3 × 10 min) with their nondominant hand requiring incrementally fast and precise pinching movements involving the thumb and index fingers. The study was designed as a semirandomized crossover study where the participants attended one practice‐ and one control session. Before and after each session electrophysiological recordings were obtained during three blocks of 50 precision pinch movements in a standardized setup resembling the practiced task. Data recorded during movements included electroencephalographic (EEG) activity from primary motor cortex and electromyographic (EMG) activity from first dorsal interosseous (FDI) and abductor pollicis brevis (APB) muscles. Changes in the corticospinal drive were evaluated from coupling in the frequency domain (coherence) between EEG–EMG and EMG–EMG activity. Following motor practice performance improved significantly and a significant increase in EEG‐EMG_APB_ and EMG_APB_‐EMG_FDI_ coherence in the beta band (15–30 Hz) was observed. No changes were observed after the control session. Our results show that tablet‐based motor practice is associated with changes in the common corticospinal drive to spinal motoneurons involved in manual dexterity. Tablet‐based motor practice may be a motivating training tool for stroke patients who struggle with loss of dexterity.

## Introduction

Manual dexterity is closely related to a well‐developed corticospinal (CS) system (Lemon and Griffiths 2005) and damage to this descending pathway is widely assumed to be the major cause of impaired dexterity after subcortical stroke (Porter and Lemon 1993). The importance of this pathway in dexterious function have been demonstrated in lesion studies performed in monkeys where a severe deficit in precise finger movements is observed following damage to the CS tract (Lawrence and Kuypers 1968). The primary motor cortex is the major contributor to the CS tract in primates (Lemon 2008) and a subset of these CS projections makes direct, cortico‐motoneuronal (CM) connections with spinal motoneurons (Porter and Lemon 1993; Lemon 2008). These direct connections operate in parallel with more indirect connections, possibly by adding the final spatiotemporal excitation patterns that would induce appropriate levels of motoneuronal recruitment and discharge (Lemon et al. 2004). In monkeys, it has been demonstrated that during relatively independent finger movements, such as the precision grip, the CS neurons, including the direct CM connections, are specifically recruited (Bennett and Lemon 1996).

Estimates of the CS drive to motoneurons have been noninvasively derived during voluntary movement from coherence analysis in human subjects (Farmer et al. 1993; Conway et al. 1995). The coherence spectrum between two signals provides an estimate of the coupling between the two signals in the frequency domain (Farmer et al. 1993; Halliday et al. 1995). Thus, coherence between cortical activity recorded by electroencephalography (EEG) and muscle activity recorded by electromyography (EMG) reflect the output of the motor cortex and its transmission to the spinal motoneurones through the CS tract during muscle activation (Halliday et al. 1998). Coherence analysis of surface EMG within and between muscles provides a complementary means of measuring and detecting changes in the CS input (Halliday et al. 2003; Norton and Gorassini 2006; Farmer et al. 2007). Adaptations in coherence have been shown to take place during a fine‐motor task that requires a high degree of attention and precision (Kristeva‐Feige et al. 2002) and to be influenced by practice (Perez et al. 2006; Mendez‐Balbuena et al. 2012).

During the last decade, the use of smartphones and tablets, which require a high level of manual dexterity, has exploded (Ofcom, 2015). Clearly the widespread and frequent use of these devices by people demonstrates the extent of its influence. Yet, little is known on which possibilities the use of touch screens offer for practice‐related improvements in manual dexterity and the functional organization of the brain. The requirement of repetitive accurate finger movements in order to navigate these devices relies on effective CS control of the finger muscles and we therefore hypothesized that CS drive to finger muscles is optimized by specific tablet‐based practice. Previous studies have demonstrated that both model‐free motor practice (Classen et al. 1998; Muellbacher et al. 2001) and practice of a novel and specific fine‐motor skill induces rapid plastic changes in the primary motor cortex (M1) (Jensen et al. 2005; Hamada et al. 2014). In this study, we investigate whether tablet‐based motor practice employing a specific application with requirements for accurate finger movements changes the CS drive measured by the coupling in the frequency domains (coherence) between EEG–EMG and EMG–EMG activity.

## Materials & Methods

### Participants

Sixteen healthy females (mean age 24, range 21–29 years) participated in the study. Only females were included as this experiment is part of a research project that focus investigation in women (Copenhagen Women Study, www.cws.ku.dk). All participants had a right hand preference as determined by a handedness questionnaire (Oldfield 1971) and all except one participant were using a tablet on a daily basis with their dominant hand. None of the participants had any history of neurological or psychiatric disorder. All of them gave their informed consent to the experimental procedure, which was approved by the ethics committee for the Greater Copenhagen area. The study was performed in accordance with the Declaration of Helsinki.

### Experimental protocol

**Figure 1:**
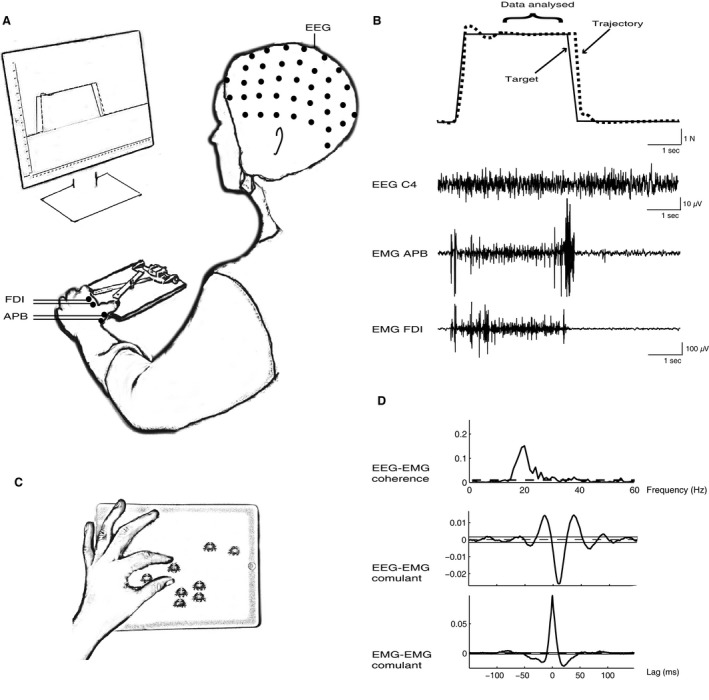
Experimental setup. (A) Participants were seated in front of a computer screen with their left forearms resting on a table and with a spring‐loaded lever placed between the thumb and index finger (B) illustrates one trial. The black line demonstrates the target consisting of a fixed ramp and the dashed line demonstrates the performance of a single participant. The horizontal curly bracket illustrates the steady hold phase of which were used for the later calculation of coherence. Raw EEG data from right sensorimotor cortex is shown together with raw EMG recordings from left APB and FDI from a single participant during one trial. (C) Illustrates the game on the tablet computer. Participants had to “pinch” crabs using the thumb and index finger to make them disappear. (D) The calculated coherence from the steady hold phase in 150 trials is shown from a single participant as well as the cumulant density function for EEG‐EMG and EMG‐EMG.

The experimental protocol was designed as a randomized crossover study for which participants attended a session involving tablet‐based motor practice and one control session. The same standardized task was performed twice at both sessions (pre and postintervention) to test coherence. This task was designed to resemble the task practiced on the tablet, but under controlled circumstances and at fixed, slower pace. The task was also chosen to optimize the assessment of CM coherence, as coherence is abolished during movement and is greater during steady periods of contraction (Kilner et al. 1999). The participants sat upright on an adjustable chair with their forearms resting comfortably on a table. The participants were instructed to control a spring‐loaded lever placed in front of them with the thumb and index finger of their nondominant (left) hand (Fig. 1A). Visual feedback of the generated force was provided on a computer screen that also contained a target consisting of a fixed ramp (Fig. 1B). The participants were asked to track the target, as accurately as possible, by applying force to the levers. A cursor moved automatically across the screen from left to right at a constant velocity and force applied to the levers moved the cursor upward. The force level of the ramp plateau (*y*‐axis) was set to 5.5 N lasting 3 sec followed by 3 sec of rest. The force was measured with a load cell (UU2‐K30, Dacell, Korea). Participants performed three blocks of 50 trials with 60 s of rest between the blocks. Before recording, participants were acquainted with the setup and trained to control the lever. The trials were recorded by Signal software (CED, Cambridge, UK) and stored for later analysis.

In the session involving tablet‐based motor practice participants were instructed to practice a game in the application Dexteria, called *Pinch it* (BinaryLabs, www.dexteria.net), with their nondominant hand for 3 × 10 min with 2 min of rest in between. We chose to use the application Dexteria because it is designed to practice dexterity and developed in collaboration with physiotherapists and because we found that it is a well‐developed application for the purpose. The application takes advantage of the multitouch interface and it applies relevant concepts such as repetition, intensity and task‐oriented training. We have not been involved in developing the application and we do not have any interest in promoting this application. The nondominant hand was selected because we expected the participants to be more naive to tablet‐based motor practice involving the nondominant hand. The game consisted of 10 levels that required incrementally precise and fast squeezing of the thumb and index finger in order to catch crabs and make them disappear. Crabs were stationary in lower levels, begin to move around in mid‐levels, turn red and multiply if pinched when red in higher levels, and move extremely rapidly while also turning red in highest levels. When the participants completed 10 levels (1 round) they were instructed to start from level one again and complete as many rounds as possible. After the tablet‐based practice, The Intrinsic Motivation Inventory (IMI) was administered to all participants. IMI is a multidimensional questionnaire structured into various subscales intended to assess participants’ subjective experience related to a target activity in laboratory experiments (Plant and Ryan 1985). In this study, the IMI was used to assess participant's interest/enjoyment, perceived competence and effort while performing the tablet‐based practice. Scores approaching seven in each subscale represent positive values in terms of motivation. In the control session, participants were instructed to read in a book in the intermediate (40 min) period between pre‐ and postmeasurements. We chose a control session without activity in the involved finger muscles because previously studies have observed no change in CM coherence after a control session consisting of simple movements without a learning objective (Perez et al. 2006; Geertsen et al. 2013). Furthermore, it is beyond the scope of this article to compare tablet‐based practice with other potential interventions. The participants were not allowed to move around or use a smartphone or a tablet.

### Electrophysiological measurements

Electroencephalographic (EEG) and electromyographic (EMG) activity were recorded from 64 and 4 channels, respectively, (ActiveTwo, BioSemi, Amsterdam, the Netherlands) using acquisition software ActiView (version 6.05) during pre and postmeasurements (Fig. 1). Active EEG electrodes were mounted in a headcap (headcap BioSemi, the Netherlands). Two pairs of bipolar active EMG electrodes were placed on the nondominant hand over first dorsal interosseous muscle (FDI) and abductor pollicis brevis muscle (APB) (interelectrode distance, 1.5 cm). As per BioSemi's design, the ground electrode during acquisition was formed by the Common Mode Sense active electrode and the Driven Right Leg passive electrode. Offset values were kept below ± 25 microV. Recordings were set to AC and filtered in the frequency range of 0.16–100 Hz, sampled at 2048 Hz and stored on a PC for off‐line analysis.

### Analyses and statistics

All EEG and EMG data were analyzed offline using Matlab R2013a (MathWorks, MA), with the toolbox EEGLAB v13.2.1 (Swartz Center for Computational Neuroscience; http://sccn.ucsd.edu/eeglab/). All files were imported to EEGLAB, 5 Hz high‐pass and 1000 Hz low‐pass filters were applied and data were re‐referenced to average reference. CM coherence in the 15–30 Hz frequency band is abolished during movement and is greater during steady periods of contraction (Kilner et al. 1999). Therefore, measurements were taken while the participants performed a tonic pinch grip. The interval between 0.85 and 2.85 sec after ramp onset showed the most stable force production across participants and was used for further analysis of coherence (displayed as the horizontal curly bracket in Figure 1B). The procedures for calculation of coherence between two signals were carried out using the methods set out in details by Halliday et al. (1995). Before undertaking further analysis, the EMG signals were full wave rectified. This approach has been shown to maximize the information regarding timing of motor unit action potentials (MUAP) while suppressing information regarding MUAP waveform shape (Halliday and Farmer 2010; Boonstra and Breakspear 2012). This procedure is particular valid in low force contractions where it clearly improves detection of motor unit coherence in the beta‐frequency band(Ward et al. 2013). As a preprocessing step before undertaking population analysis of the data, the EEG channel placed at the right M1 (C4) and the rectified EMG signals were normalized to have unit variance (Halliday and Rosenberg 2000). Normalized signals are assumed to be realizations of stationary zero mean time series, denoted by *x* and *y*. The single EEG electrode (C4) above the right M1 had the highest EEG‐EMG coherence in the first subjects and was selected for further analysis. Power spectra were constructed from sections of data taken at a fixed offset time with respect to a trigger point (0.85 sec after start of pinch grip) in each trial. Estimates of the power spectra were constructed by averaging periodograms across all trials*. f*_xx_ *(λ)* and *f*_yy_ *(λ)* represent the Fourier transforms of processes *x*, and *y*, at frequency *λ*. The cross spectrum between *x* and *y* is denoted by *f*_xy_ *(λ),* and is estimated in a similar manner. Two functions were then used to characterize the signals’ correlation structure: coherence, *|R*_xy_ *(λ) |* ^*2*^ and cumulant density, *q*_xy_ *(u)*. Coherence estimates are bounded measures of association defined over the range [0, 1]. The cumulant density provides an unbounded time‐domain representation of the EEG‐EMG or EMG‐EMG correlation structure analogous to the motor unit cross‐correlogram (Halliday et al. 1995). For the present data, coherence estimates provide a measure of the fraction of the activity in the two surface EMG signals (EMG_APB,_ EMG_FDI_) at any given frequency that can be predicted by the activity in the EEG signal measured at the contralateral M1 as well as the fraction of activity in the EMG_APB_ signal that can be predicted by the activity in the EMG_FDI_ signal. In this way, the coherence in this particular experiment provides an estimate of the cortical input to the two muscles (CM coherence) as well as an estimate for the common input to the two muscles [inter‐muscular (IM) coherence]. In order to summarize the correlation structure across participants, the individual coherence and cumulant density estimates were pooled providing a single time or frequency domain measure (Amjad et al. 1997). The interpretation of pooled estimates of spectrum, coherence, and cumulant is similar to those obtained for individual records, except any inferences related to the population as a whole.

Statistical inference regarding the difference of coherence between two independent estimates can be made using the *χ* ^2^ extended difference of coherence test (Amjad et al. 1997). Significance is assessed through inclusion of an upper 95% confidence limit. Estimates of pooled coherence were used to summarize the correlation structure before and after intervention in both conditions. In addition to pooled statistics, the cumulated sum of the logarithmic values of EEG‐EMG and EMG‐EMG coherence in the beta (15–30 Hz) were collected. A linear multilevel regression with an unstructured repeated covariance type was used to investigate the effect of tablet‐based practice on coherence (with session (training versus control) and time (pre versus post) as factors). Bonferroni's post hoc test was performed on significant comparisons. A one‐way repeated‐measures analysis of variance (ANOVA) with Bonferroni correction was performed to investigate the practice effect measured as number of crabs pinched in the first, second, and last 10 minutes of training. Results are presented in means ± SD in text and as means ± SE in figures. A two‐tailed Pearson's correlation analysis was performed to test correlation between motor performance progression and the magnitude of change in coherence after practice. Statistical analyses were performed with SPSS 22.0 for Mac (IBM, Armonk, NY).

## Results

### Change in motor performance and motivation scores

**Figure 2:**
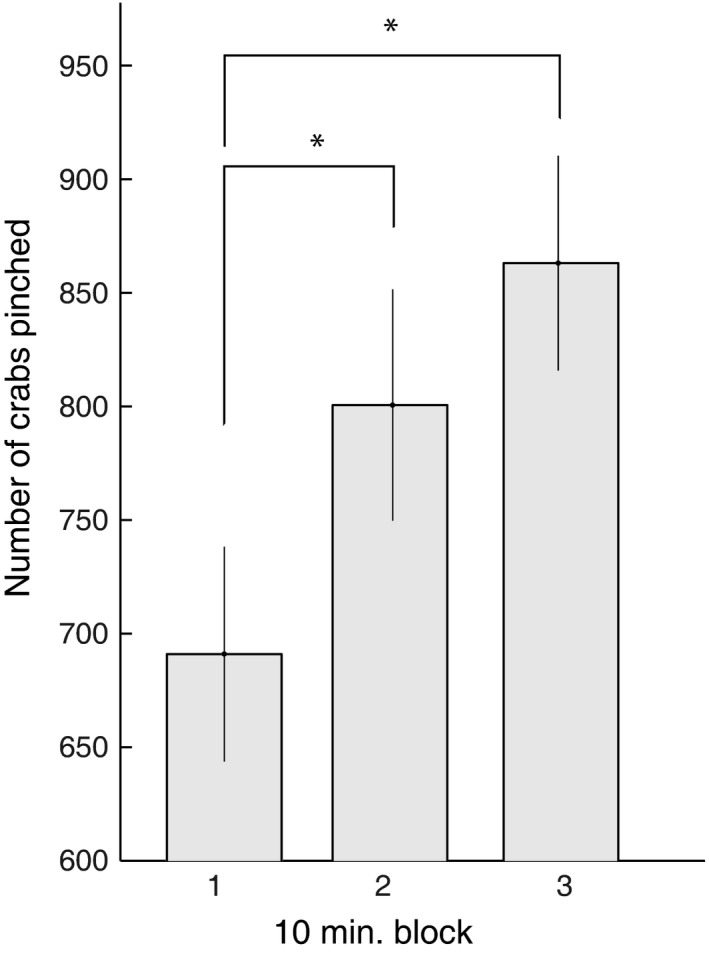
Bar graph demonstrating number of crabs pinched in the first, second and third 10 min. Block of tablet‐based practice (*n* = 16). Error bars indicate standard errors of the mean (*P < 0.001).

The participants showed a significant improvement in number of pinched crabs from 691 ± 189 crabs in the first 10 min to 801 ± 204 crabs in the next 10 min (*P* < 0.001) to 863 ± 189 crabs in the last 10 min (*P* < 0.001) (Fig. 2).

Participants reported high levels of interest and enjoyment in relation to the tablet‐based practice (4.64 ± 1.59) and perceived competence (5.39 ± 1.11) as well as effort (6.61 ± 0.59).

### Changes in coherence

**Figure 3:**
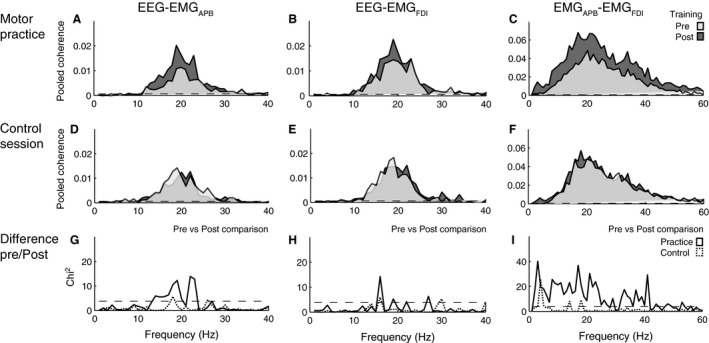
Pooled coherence data from EEG‐EMG_APB_, EEG‐EMG_FDI_ and EMG_APB_‐EMG_FDI_ from all 16 participants. Coherence is calculated from 2 sec hold phase in 150 trials. A, B and C shows pooled coherence pre (light gray) and post (dark gray) practice. D, E and F shows pooled coherence pre (light gray) and post (dark gray) control. G, H and I show the *χ* ^2^ extended test for differences of coherence between pre and post in the practice session (solid line) and between pre and post in the control session (dashed line). Note that the *χ* ^2^ values give the statistical differences between the measurements and that peak values of *χ* ^2^ values may indicate both an increase and decrease in coherence. It is therefore not possible to determine from the bottom line of graphs which of the measurements was the largest. This can only be determined from the two above graphs. The dashed horizontal lines in all plots denote the 95% confidence limits (*χ* ^2^ (0.05–1) = 3.84).

Data show an increase in the coupling of CM and IM coherence in the frequency ranges 15–30 Hz after tablet‐based practice. This result was clear from visual inspection of individual coherence data from pre and postmeasurements in the two sessions, and these conclusions were confirmed through application of pooled data analysis, including the extended difference of coherence (*χ* ^2^) test. The pooled coherence data from all 16 participants and the extended *χ* ^2^ difference of coherence are shown in Figure 3. Figure 3A–C shows coherence before and after the practice session, whereas Figure 3D–F shows the estimates from the control session. The difference in coherence estimates resulting from tablet‐based motor practice evaluated using the *χ* ^2^ test is shown for each frequency comparison in Figure 3G–I. The coherence estimate between EEG‐EMG_APB_ increased after the practice session, but not after the control session (Fig. 3A and D). The *χ* ^2^ test confirmed a significant difference in the coherence in the frequency band 15–25 Hz between pre and postpractice data indicating a training related increase in EEG‐EMG_APB_ in beta band frequencies (Fig. 3G). In contrast *χ* ^2^ test revealed no differences between recordings obtained in the control session (Fig. 3G). The pooled results for EEG‐EMG_FDI_ shows a small increase in coherence following tablet‐based motor practice in the frequency range 15–21 Hz (Fig. 3B), but not after the control session (Fig. 3E). The extended *χ* ^2^ test (Fig. 3H) revealed a narrow significant difference in the coherence in the frequency band of 15–17 Hz between pre and postpractice data and no differences between recordings obtained in the control session. The coherence estimate between pooled IM coherence data for EMG_APB_‐EMG_FDI_ increased following tablet‐based motor practice (Fig. 3C) compared to the control session (Fig. 3F) in a broad frequency range from approximately 10–40 Hz. The extended *χ* ^2^ difference of coherence test confirmed a significant coherence difference in a similar broad frequency band between pre and postpractice data and no differences between recordings obtained in the control session, indicating a training related increase in IM coherence (Fig. 3I).

**Figure 4:**
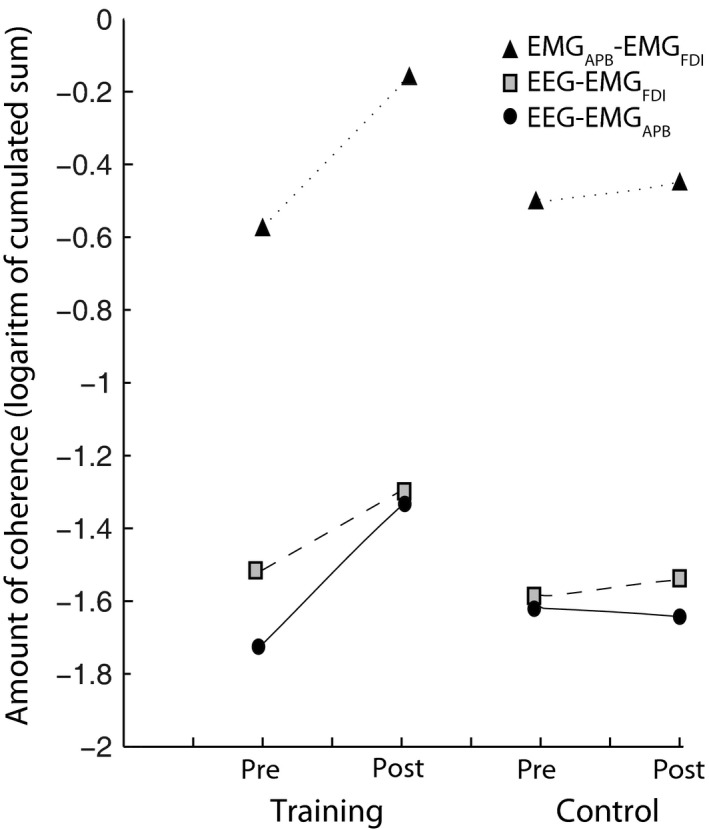
The cumulated sum of the logarithmic values of coherence was estimated for all participants in the EEG‐EMG_APB_, EEG‐EMG_FDI_ and EMG_APB_‐EMG_FDI_ in the beta (15–30 Hz) band. The respective pre and post mean values are plotted for the practice session (left panel) and for the control session (right panel).

To further quantify changes in the CM and IM coherence, the logarithmic value of the sum of coherence in the beta band (15–30 Hz) was calculated and compared between pre and postmeasurements in the two sessions (Fig. 4). A linear multilevel regression analysis showed a significant interaction between session (practice versus control) and time (pre vs. post) in the beta band in EEG‐EMG_APB_ (*F* = 7.288; *P* = 0.016) and post hoc analysis revealed significant increase in coherence after practice when compared with before practice (*P* = 0.0004). No interaction was found in the beta band (15–30 Hz) in EEG‐EMG_FDI_ (*F* = 1.740; *P* = 0.206) between sessions and time. However, there were a significant interaction between sessions and time in the beta band in EMG_APB_‐EMG_FDI_ (*F* = 4.611; *P* = 0.047) and post hoc analysis revealed a significant increase incoherence after practice compared to before practice (*P* = 0.008). Pearson correlation analysis showed a moderate correlation between performance progression in the tablet game and the magnitude of enhancement of EEG‐EMG_APB_ coherence after the tablet‐based practice session (*r* = 0.477, *P* = 0.062) and a moderate correlation to EEG‐EMG_FDI_ (*r* = 0.432, *P* = 0.094) and a weak correlation to EMG_APB_‐EMG_FDI_ (*r* = 0.175, *P* = 0.517).

Pearson correlation analysis showed a strong significant correlation between the enhancement in EEG–EMG_APB_ coherence and EMG_APB_‐EMG_FDI_ coherence after practice (*r* = 0.61, *P* = 0.012) and between EEG‐EMG_APB_ coherence and EEG‐EMG_FDI_ coherence after practice (*r* = 0.629, *P* = 0.009), but not between EEG‐EMG_FDI_ coherence and EMG_APB_‐EMG_FDI_ coherence (*r* = 0.159, *P* = 0.556).

**Figure 5:**
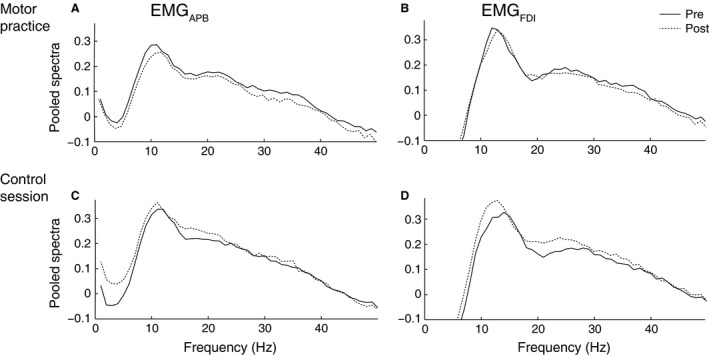
Average across trials and participants (pooled) power spectra (1–50 Hz) of EMG_APB_ and EMG_FDI_. A and C, the pooled spectra from EMG_APB_ before (solid line) and after (dashed line) the practice (A) and control session (C). B and D, the pooled power spectra from EMG_FDI_ before (solid line) and after (dashed line) practice (B) and control session (D). The power spectra are constructed of the 2 sec hold phase in the 150 trials.

The pooled cumulant density function calculated from EEG‐EMG_APB_ and EEG‐EMG_FDI_ before and after either the practice or the control session was characterized by a central peak observed at a lag around 10–12 ms, whereas EMG_APB_‐EMG_FDI_ peaked in 0 ms (Fig. 1D). The pooled EMG power spectra data in the practice session is shown in Figure 5A–B and the control session in Figure 5C–D. In both EMG_APB_ and EMG_FDI_ power spectra characteristic peaks around 10–15 and 20–25 Hz were observed.

## Discussion

To our knowledge this is the first study to demonstrate that 30 min of fine‐motor practice requiring accurate and fast finger movements with incremental task difficulty results in changes in the corticomuscular coherence. The magnitude of CM and IM coherence in the frequency range 15–30 Hz increased following tablet‐based motor practice, whereas no change in coherence was observed after a control session. We propose that the changes in coherence reflect plastic changes in the CS drive to the spinal motoneurones relating to optimized task performance.

### What causes CM and IM?

CM coherence reflects coupled beta band oscillations in the muscles and the contralateral motor cortex, which in all likelihood are generated by activity in the cortico‐motoneuronal pathway (Conway et al. 1995; Mima and Hallett 1999). CM coherence may be disrupted or reset by stimulation of the motor cortex (Hansen and Nielsen 2004) and it is absent or highly reduced in subjects with stroke (Rossiter et al. 2012). Sensory feedback mechanisms likely contribute to the generation and magnitude of CM coherence, as manipulation of sensory input modulates the coherence (Hansen and Nielsen 2004; Riddle and Baker 2005; Stancak et al. 2005) and since strong beta‐band oscillations have been demonstrated in the somatosensory cortex, which are synchronized with those in motor cortex (Witham and Baker 2007; Witham et al. 2007).

IM coherence in the beta band is related to CM coherence (Kilner et al. 1999; Grosse et al. 2003; Farmer et al. 2007) (Fig. 3) and appears to reflect the common oscillatory drive to the motor units from branches of corticospinal tract fibers (Farmer et al. 1993; Farmer 1998; Hansen and Nielsen 2004).

### Changes in CM and IM coherence with motor practice

Cross‐sectional studies have indicated that the amount of CM and IM coherence may depend on the functional abilities of the individual (Semmler et al. 2004; Ushiyama et al. 2010), but this is challenged by training studies (Perez et al. 2006; Geertsen et al. 2013). Similar to this study, changes in coherence have been observed with improvements in motor performance (Perez et al. 2006; Geertsen et al. 2013), but disappear again within minutes following a single training bout, despite maintained motor performance abilities. In this study, we did not obtain a second delayed measure of coherence following motor practice, as the test was very time consuming and involved considerable mental and physical effort. Furthermore, additional tests could have influenced coherence measures, for example, via fatigue. However, half of the participants were randomized to the control trial following the intervention trial and we could therefore compare their baseline measure in the control trial to their postintervention measure. The changes in coherence seen following the practice were not maintained at the second visit suggesting that the changes in coherence are related to processes immediately after motor practice (data not shown). This was also concluded by Perez et al. and Geertsen et al. (Perez et al. 2006; Geertsen et al. 2013) and in this sense the changes in coherence may resemble what has been observed for excitability or representational changes in the motor cortex accompanying skill acquisition (Perez et al. 2004; Jensen et al. 2005; Pascual‐Leone et al. 2005). It would seem plausible that the mechanisms responsible for changes in M1 excitability and the reorganization of the representational maps would also influence the discharge of the corticospinal tract neurons and produce the observed changes in coherence. It has been demonstrated that intracortial inhibition decreases in relation to the increased corticospinal excitability observed following motor learning (Liepert et al. 1998; Classen et al. 1999; Perez et al. 2004), which suggests that changes in the cortical neuronal circuitry may be involved in changes in cortical representations following extended motor practice. Such changes in corticospinal excitability may also be involved in the increased CM coherence, as both inhibitory and facilitatory local circuitries likely influence the oscillatory properties of the CS cells significantly (Baker and Baker 2003; Hansen and Nielsen 2004). Similar to what has been found for cortical representational maps, repeated practice of the task may result in more lasting and possibly permanent changes in CM and IM coherence, but this has to our knowledge never been investigated and in any case not in relation to tablet‐based motor practice.

We chose a test setup, that is, a transfer task (squeezing of index and thumb against spring resistance) in this study, which resembled the practice task as much as possible in order to facilitate the relation between changes in behavior and changes in coherence. Furthermore, the test task was performed dynamically, which is novel compared to most other studies that measure coherence. We observed a moderate correlation between the progression in performance and coherence suggesting that the participants who displayed behavioral improvements were also, at least to a certain extent, the participants who had the largest change in coherence. This opens the possibility as also suggested by Perez et al. 2006 that the changes in coherence reflect changes in the corticospinal system that are involved and possibly even responsible for the acquisition of the task (Perez et al. 2006).

Both motor skill acquisition and coherence could also relate to the attentional demands of the task and the attention of the participant. Rosenkranz and Rothwell (2006) showed that the pattern of reorganization produced by sustained sensory input depends not only on the participants’ attention but also on the spatial focus of their attention on the body surface involved (Rosenkranz and Rothwell 2006). In this study, the participants had to maintain attention on the pinch movements in order to keep track of the trace during the standardized task and toward their fingers in order to enhance motor performance during motor practice. Thus, these attentional demands and the requirements to integrate proprioceptive and visual input may also contribute to the change in coherence.

In this study, while we did see a difference in EEG‐EMG_APB_ CM coherence, there was no difference found in EEG‐EMG_FDI_. Participants may have squeezed and released the levers with a more dynamic use of the index finger (T. Jensen and L. H. Larsen, pers. obs.), although they were told to use both fingers equally. CM coherence is enhanced during a sustained contraction and attenuated by movement (Kilner et al. 1999). A more dynamic use of the index finger in the precision grip task may therefore have eliminated changes in EEG‐EMG_FDI_ coherence. It should also be considered that optimization of task performance most likely involved other joints and muscles than the finger muscles for which coherence was estimated, for example, in the shoulder and forearm.

### Clinical perspectives

The ability to perform a precision grip and other independent finger movements is an essential part of our daily life activities. In stroke, which is the main cause of serious and long‐term disability in the world (WHO, 2002), loss of both force and dexterity in the upper limb is one of the most common and most devastating consequences. Treatment of these patients relies on intensive rehabilitation, where compliance is made difficult by the repetitive nature of traditional therapies (Langhorne et al. 2009). It is therefore not surprising that current doses of task‐specific practice have been shown to be inadequate to drive the neural changes (Lang et al. 2009). Hundreds of daily repetitions of upper extremity functional movement are most likely needed during rehabilitation to optimally promote function post stroke (Lang et al. 2009). Tablet‐based motor practice involving specialized apps might be a potential motivating and effective training tool to practice manual dexterity at home and enhance neural plasticity. In this study, the participants practice with a specific application that required incrementally precise and fast squeezing of the thumb and index finger. They performed more than 2000 pinching movements for 30 min, and assessed from the IMI interest/enjoyment score, without being bored. This amount is not comparable to what stroke patients can manage, but it is noteworthy that the participants found the game‐based motor practice entertaining and persistently tried to improve assessed based on the high scores of perceived competence and effort in IMI. In addition, the specific application required constant attention to the finger movements in order to enhance performance and this might be relevant in order to optimize the outcome of rehabilitation practice (Rosenkranz and Rothwell 2006). Thus, recent progresses in touch screen technology offer possibilities, which are important for rehabilitation, including the provision of multisensory (visual, auditory and tactile) feedback, as well as repetition, intensity, and task‐oriented practice (Larsen et al. 2013). Thus, tablet computers combined with specialized apps could potentially be used to practice manual dexterity in stroke patients with a paretic hand.

## Conclusion

With this study, we have demonstrated corticospinal plasticity in relation to motor practice requiring fast and accurate pinch grip using a touchscreen. Motor performance improved during tablet‐based practice. We propose that the changes in cortico‐motoneuronal and intermuscular coherence reflect changes in the corticospinal drive to the spinal motoneurones as part of the binding process of new information between cortex and the muscle associated with behavioral improvements. This is of potential interest for rehabilitation of manual dexterity after stroke.

## Conflict of Interests

The authors declare no competing financial interests.
